# A Smartphone-Based Cursor Position System in Cross-Device Interaction Using Machine Learning Techniques

**DOI:** 10.3390/s21051665

**Published:** 2021-02-28

**Authors:** Juechen Yang, Jun Kong, Chunying Zhao

**Affiliations:** 1 Department of Computer Science, North Dakota State University, Fargo, ND 58105, USA; juechen.yang@ndsu.edu (J.Y.); jun.kong@ndsu.edu (J.K.); 2 School of Computer Sciences, Western Illinois University, Macomb, IL 61455, USA

**Keywords:** cross-device interaction, motion detection, gestural interaction, large display, mobile device

## Abstract

The use of mobile devices, especially smartphones, has become popular in recent years. There is an increasing need for cross-device interaction techniques that seamlessly integrate mobile devices and large display devices together. This paper develops a novel cross-device cursor position system that maps a mobile device’s movement on a flat surface to a cursor’s movement on a large display. The system allows a user to directly manipulate objects on a large display device through a mobile device and supports seamless cross-device data sharing without physical distance restrictions. To achieve this, we utilize sound localization to initialize the mobile device position as the starting location of a cursor on the large screen. Then, the mobile device’s movement is detected through an accelerometer and is accordingly translated to the cursor’s movement on the large display using machine learning models. In total, 63 features and 10 classifiers were employed to construct the machine learning models for movement detection. The evaluation results have demonstrated that three classifiers, in particular, gradient boosting, linear discriminant analysis (LDA), and naïve Bayes, are suitable for detecting the movement of a mobile device.

## 1. Introduction

With the rapid development of mobile computing technology, mobile devices, especially smartphones, have become popular in recent decades. According to the Pew Research Center, 81% of Americans own smartphone and roughly half of U.S adults own tablet devices in 2020 [1]. The popularity of mobile devices creates an increasing need for cross-device operations.

Large display devices have been widely deployed in diverse environments in our daily lives for a better viewing experience. Some of the large screens serve as broadcast platforms that raise no interest from bypassers [2]. They are static and have barely any user-triggered interaction. The size of the screen makes it inaccessible for viewers to interact with the contents on the screen. Viewers can only passively receive information from the screen. The use of these public displays also raises health concerns due to the high-frequency use of contaminated input hardware, such as the touchscreen and the game handler. Direct methods of interaction present numerous issues that cross-device interactions could solve. As mobile devices are widely used, the idea of using mobile devices to interact with large screens has been proposed in recent decades. 

Many techniques have been proposed for cross-device interactions. Ikematsu and Siio [3] created a “drag-and-drop” style of interaction that allows the user to transfer data objects between two touchable devices. Gradual Engagement [4] can automatically detect transferrable data objects between devices that are physically close to each other. To transfer the data, this application requires the user to drag the detected data object to the display zone on the destination screen. These two approaches are only viable when all the screen regions of the large display device are physically accessible. Current trends, however, have indicated that the size of large display devices will continue to increase because a larger target display leads to more efficient and effective interactions [5]. If the size of a large display device is extremely large and not all the regions are physically accessible, it could be necessary to build a seamless connection between a mobile device and a large display device. To access unreachable large display screens, Torch projector [6] uses a “pick and drop” style that allows users to pick the view on a mobile device and project it on a large display at a distance. Then the user can interact with the object in the view from the mobile device. In their work, the large display receives information from multiple mobile devices as a remote and touchable projector.

With the popularity of smart phones and tablets, users often switch between different devices, which makes it challenging to share information across devices in a collaborative environment. This paper presents a machine-learning based approach that seamlessly shares information between a large display and a mobile device and supports simultaneous multi-user interaction with the large display. Specifically speaking, a user moves his/her mobile device on top of a flat surface. The movement that is detected by analyzing accelerometer data indicates the change of a user’s focus on a large display. Based on the movement, the contents displayed on the mobile device is updated accordingly. By clicking on an object on the mobile device, the corresponding object is transferred from the large display to the mobile device. In such an interaction style, a mobile device can function both as a cursor for changing a user’s focus and as a personal workspace for remote manipulation. 

To initialize the cursor position on the large display, we use sound localization to determine the 2D coordinates of the cursor’s starting position on the large display screen. At the same time, a resolution conversion has been applied to ensure that the size of the area cut from the large display is physically equal to the size of the mobile device. When the user interacts with the large display using the mobile device, the cursor’s new position on a large display will be updated accordingly. 

Detecting the motion of a mobile device is crucial in the cross-device interaction because we use the mobile device as a cursor on the large screen device. To achieve this, we collect motion data of the mobile device from the built-in accelerometer. Accelerometers have been used in detecting human daily activities [7,8], such as falls, gait, and gesture detection. In our approach we detect the movement of mobile devices, so we collect more fine-grained motion data. A data-analysis pipeline is built for characterizing data into statistical features (mean, standard deviation, min-max difference, and power energy) and spectral features (dominant frequency and spectral energy). We have implemented both 10-folds cross-validation and a confusion matrix and applied multiple feature-selection methods in order to identify the most relevant features that contribute to movement detection. The results of this study reveal that three classifiers: gradient boosting, linear discriminant analysis (LDA), and naïve Bayes, have demonstrated high performance. Feature-selection tests indicate that features that combine speed and mean (or speed and median) can contribute the most to the recognition rate. However, performance of classification can be boosted by using features that include all vectors (acceleration, angular velocity, and speed).

Our cursor position estimation system has the following characteristics:

- **Self-contained**. Our system only uses a built-in accelerometer for detecting the movement of the mobile device without any extra hardware.
- **Intuitive**. Our system applies a natural movement gesture to directly manipulate contents on a large display. Since users are already familiar with the movement gesture on desktop interactions, our technique does not require extra training.
- **Physically Unconstrained**. Physically unconstrained typically means that the cross-device application should not require the user be physically close to the large display device. With a remote-control mechanism, multi-user participation becomes viable since the users do not need to stand close to each other in front of the large display.

In summary, we have implemented a novel cursor position estimation system for detecting the motion of a mobile device in cross-device interaction. A novel sound localization technique is used to initiate the cursor’s starting position on a large display device, which enables the initial connection between a mobile device and a large display screen. Our system is featured with a novel data collection method that avoids mislabeling for training sets when working with very sensitive data from an accelerometer. We propose a hybrid method for feature selection and evaluation. In other words, both algorithm-based and manual hypothesis-based strategies are conducted. A comprehensive study of different machine-learning algorithms and feature selection sets is conducted in the experiment. The evaluation results may help future research on improving the accuracy of motion detection in a cross-device interaction. 

Our approach is fundamentally different from vision-based approaches of movement detection. In most vision-based approaches, a camera has to be set up in advance by pointing to the workspace with an appropriate angle. In addition, if a mobile device is moving on an identical flat surface, such as a completely brown desktop, two continuous frames are too similar to detect movements. Instead, our approach uses the built-in accelerometer without needing an external camera. Therefore, our system is self-contained and can be applied to any flat surface without a pre-setup. When multiple devices are moving on a flat surface, they can interfere with each other in a vision-based analysis. On the other hand, our approach analyzes the accelerometer data from each individual device and, thus, avoids interference. Therefore, our approach is more suitable for multi-user interaction. 

The rest of the paper is organized as follows. Section 2 reviews related work. Section 3 overviews the workflow of the cursor position estimation system and explains how it works. Section 4 focuses on the cursor initialization process. Section 4 and Section 5 describe the design of the pixel-movement experiment, data collection, and data preprocessing. Section 6 and Section 7 present the machine-learning models and analyze multiple feature-selection methods. Section 8 concludes the paper and presents our future work.

## 2. Related Work

### 2.1. Interaction-Sensing Techniques

Studies of cross-device techniques and applications have been an emerging field. Many solutions have been proposed. We classified the related research based on how the interactions were made.

- Direct touch on a large display

Strohmeier [9] has introduced an interaction framework that uses the mobile device as the operational commander to initiate designated operations and to implement them through direct finger touch. For example, users can pick a color on their personal devices and can then draw a shape on the large target display using a finger motion. Schmidt et al. [10] provided a novel solution that combines the physical touch initiated from a mobile device with its orientation to indicate the target interaction region and to manipulate various operations. The restriction of this framework is that it does not allow the remote control of the target region and, thus, creates barriers to multiple users interacting with the large display simultaneously. Another project called SleeDCursor [11] is a target-region-selection application that uses a touch-based system to provide users with increased flexibility in that they can initiate the binding of a device through close-coupling (where one selects the closest device to interact with). However, users are still forced to maintain physical proximity to the large public screen in order to exchange information. Consequently, if multiple users initiate data transfers from the public screen simultaneously, there is not enough physical space for the users. All these direct-touch applications share the same requirement that the user must have physical access to the screen of the target large display device. To address this problem, our renovated cursor position estimation system can offer users a remote controlling experience that significantly improves the flexibility of the usage.

- Using pointing devices

Pointing devices, such as laser pointers, have been used to help the server identify the position of the mobile device. PointerPhone [12] used laser pointers and cameras on the server’s system to detect the laser-point motion and control the large display screen remotely. Another hybrid technique with a gesture-assisted, head-based coarse pointing style has been introduced in this work [13]. This technique has created predefined gesture combinations in order to trigger the pointing task, and the technique used an equipped headset to perform a precise position estimation of the point thereafter. For example, a user could initiate a tap gesture on the touchpad surface followed by a drag operation so as to activate the pointing task and enable any area of the large display to be reached with absolute precision. Nonetheless, this approach requires additional devices and cost for the user. Moreover, gesture-initiated pointing increases the complexity of manipulation. Users may have a higher chance of triggering an undesired operation. Our work uses built-in sensors, which reduces the cost and minimize the learning curve of additional devices.

- Using built-in sensors

Another category of research utilized built-in sensors, such as accelerometers and gyroscopes, to sense the mobile device’s movement. This study [14] has proposed three interaction styles to mimic the movement of the device: “tilting”, “scrolling”, and “sliding move”. Gestures “tilting” and “scrolling” were created to evaluate the motion using a built-in accelerometer that calculated the value of acceleration continuously. “Sliding move” is used to project the position. “Sliding move” is more intuitive and is similarly to the cursor action (such as moving up and down or left and right) and could be easier for users to understand and learn. Furthermore, the implementation has been renovated by means of collecting data from motion sensors (accelerometer and gyroscope) instead of a camera. In terms of pairing devices, many techniques have been developed. SyncTap [15] is collaborative pairing system for cross-device interactions that allows multiple users to pair devices with a single tap on the touchscreen. Point&Connect [16] is a technique for combining devices by leveraging the built-in microphone and acoustic signals. Yuan et al. [17] have proposed using a cross-device tracking framework to identify “same” devices in terms of users’ typing actions and then building secure cross-device communication.

- Using built-in cameras

Inspired by optical projection, virtual projection [18] uses the handheld’s built-in camera (i.e., its live video feed) to stream its video data wirelessly to the server of the large display. The server handles all video streams from clients and performs feature tracking and spatial calculation. Torch projector [6] allows users to interact with remote screens at a distance through a live video image on their mobile device. A machine serves as the environment manager to calculate the spatial relationship of the handheld device based on the change of image frames. Coordinated by the environment manger, the handheld device can drag an object from one display and drop to a target display. CamCutter, a cross-device interaction technique that allows a user to quickly select and share an application running on another screen using the camera of a handheld device [19]. This application uses computer vision algorithms and achieves a real-time synchronization between devices. These approaches highly rely on the resolution and the view of the camera. The instability of mobile device in motion (such as hand tremor) may affect the quality of camera images as well. In a multi-user environment, camera views may be blocked or affected by users nearby. 

### 2.2. Sensor-Based Motion Detection

Several studies have sought to improve the utilization and analysis of data generated from accelerometers by sensing the daily activities of people. A study by Chen et al. [20] has introduced the use of accelerometers for detecting a person’s fall. Their study has also detailed a basic workflow for parsing and filtering the data retrieved from the accelerometer. Their project has introduced and investigated features such as the sum vector, rotation angle, and slope to detect falls with a degree of both specificity and sensitivity. Furthermore, their study has noted a critical decision-making strategy: it is no longer sufficient to determine results based on the generated data by simply proposing different thresholds in making predictions. Instead, machine-learning models and algorithms should be applied to extract patterns from the observed data and to help solve complex problems.

Another fall-detection study performed by Rakhman et al. [21] has tried to detect fall-down activity through the magnitude of both the accelerometer and gyroscope and through the rotation angle of the mobile device. They have proposed an in-house algorithm to calculate all the features needed and to discover the thresholds on values for fall-down determination. Moreover, they have categorized fall activity into four subcategories, such as “fall forward” and “fall backward”, to measure the accuracy rate.

A gait-sensing study by Ferrero et al. [22] has comprehensively investigated how to sense human gaits based on the data collected from an accelerometer. Their study has introduced some crucial data-preprocessing steps, including linear interpolation, data normalization, and noise filtration. Due to the Earth’s gravitational force, it is ideal to incorporate all three dimensions’ acceleration data in an analysis. However, if a mobile device can be placed perpendicular to the ground throughout the sensing session, then the model should be adjusted to assume that only one dimension is affected by gravity. 

The above related work on motion detection of daily activities shed lights on our motion data collection using accelerometers. The major difference between daily activities and cursor movement is the time interval for data collection. The time window used in sensing daily activities is normally 1 or 2 s. We are, however, pursuing an extremely sensitive system that uses a 0.015 s time window in order to detect a device’s movement as a cursor. The movement of mobile device will determine its precise position on the large display. This requirement has imposed challenging tasks, such as preprocessing the raw data and tagging the classification samples. Our work may help future motion detection of mobile device on a large display in a cross-device interaction.

### 2.3. Cross-Platform Applications

As mobile devices get more popular in people’s daily life, numerous apps have been created serving different purposes. iOS and Android, the two major operating systems, have their own software ecosystems. In recent years, studies have focused on developing cross-platform applications. Cross-platform applications are software that can run on multiple operating systems or environments. Biørn-Hansen et al. [23] conducted a comprehensive survey and taxonomy of the research in the field of cross-platform. Rieger and Majcrzak [24] proposed an evaluation framework for cross-platform applications. Cross-platform application is still a challenge research area due to the unique development environment of each platform. 

Our work is slightly different from cross-platform applications. Cross-platform applications aims at running an application on multiple devices with different platforms, while our work currently focuses on using portable mobile devices to interact with large display screens that are hard to reach. Our system utilizes mobile devices with Android OS (client side) and Windows operating systems in the large display device (server side). Extending our system to other platforms, e.g., iOS, will increase the system’s portability. 

## 3. System Overview

**Figure 1:**
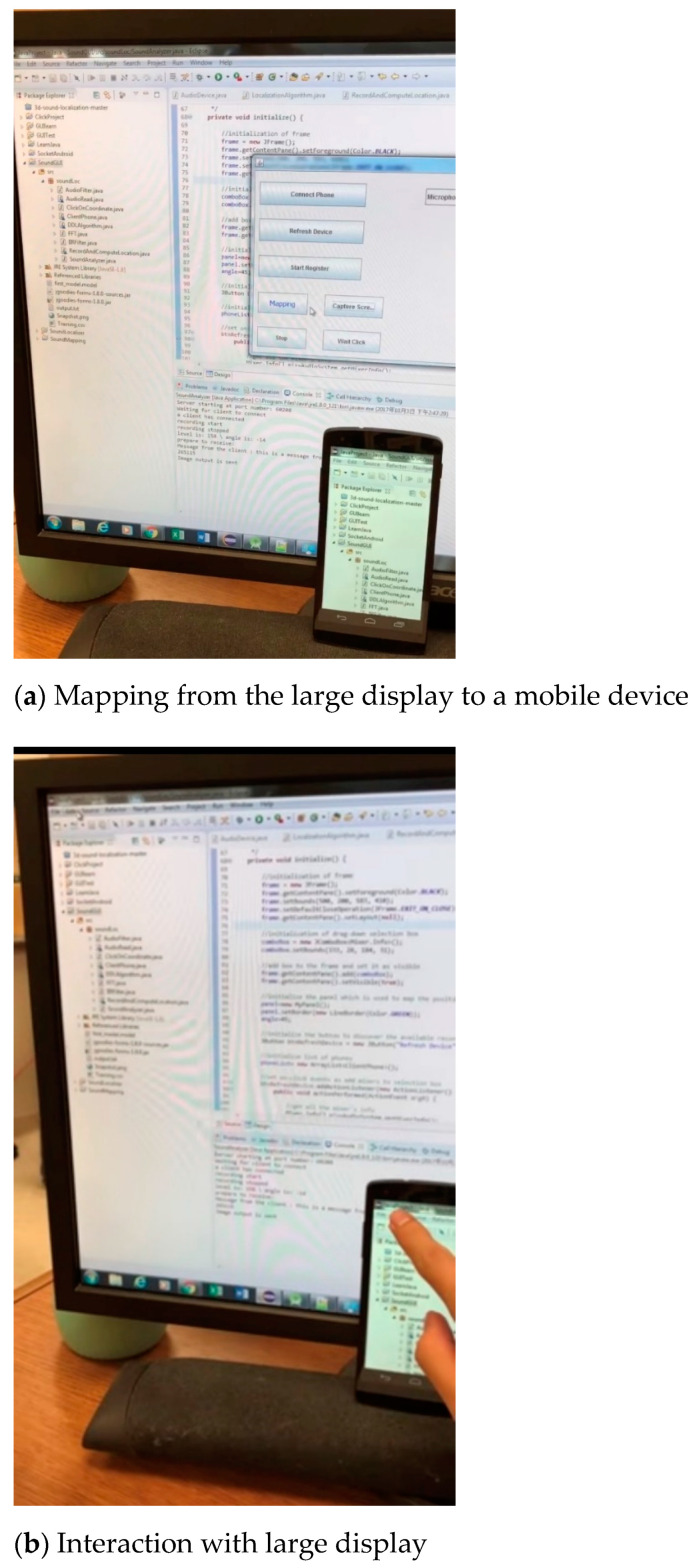

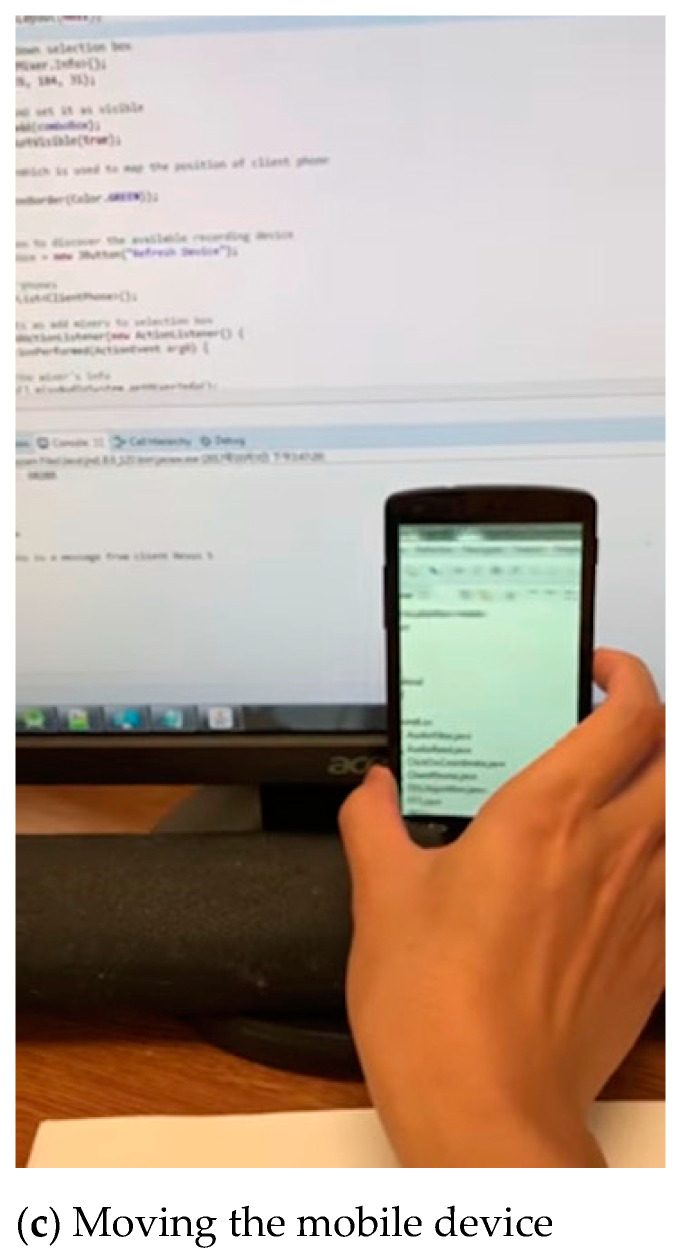
The cursor-position estimation system.

Figure 1 shows the cursor position estimation system. The system was developed on an Android platform using API level 16. The actual experiment was performed on a Samsung Galaxy S4, which is an Android mobile device. A cross-device large display server was set up on a traditional desktop device embedded with a Windows operating system. Any Java-supported desktop or device using any operating system could also serve as the large display device. An Andrea SoundMAX Superbeam array microphone (Manufactured by Andrea Electronics Corporation, Bohemia, NY, US) was used in the desktop to enable sound localization. 

In Figure 1a, the content of on the large screen is mapped to the interface of the smartphone. The smartphone uses the desk surface as the 2D flat surface. When it moves, the contents from the large screen will be updated on the smartphone accordingly. The user can easily interact with the large screen from her/his smartphone as shown in Figure 1b,c shows the user moves the mobile device and the contents are updated. The cursor position estimation system consists two major steps. First, it initiates the starting position of a cursor on a large display through sound localization. Second, a movement translation system transfers the mobile device’s movement on a flat surface to the cursor’s movement on a large screen through machine learning models.

**Figure 2:**
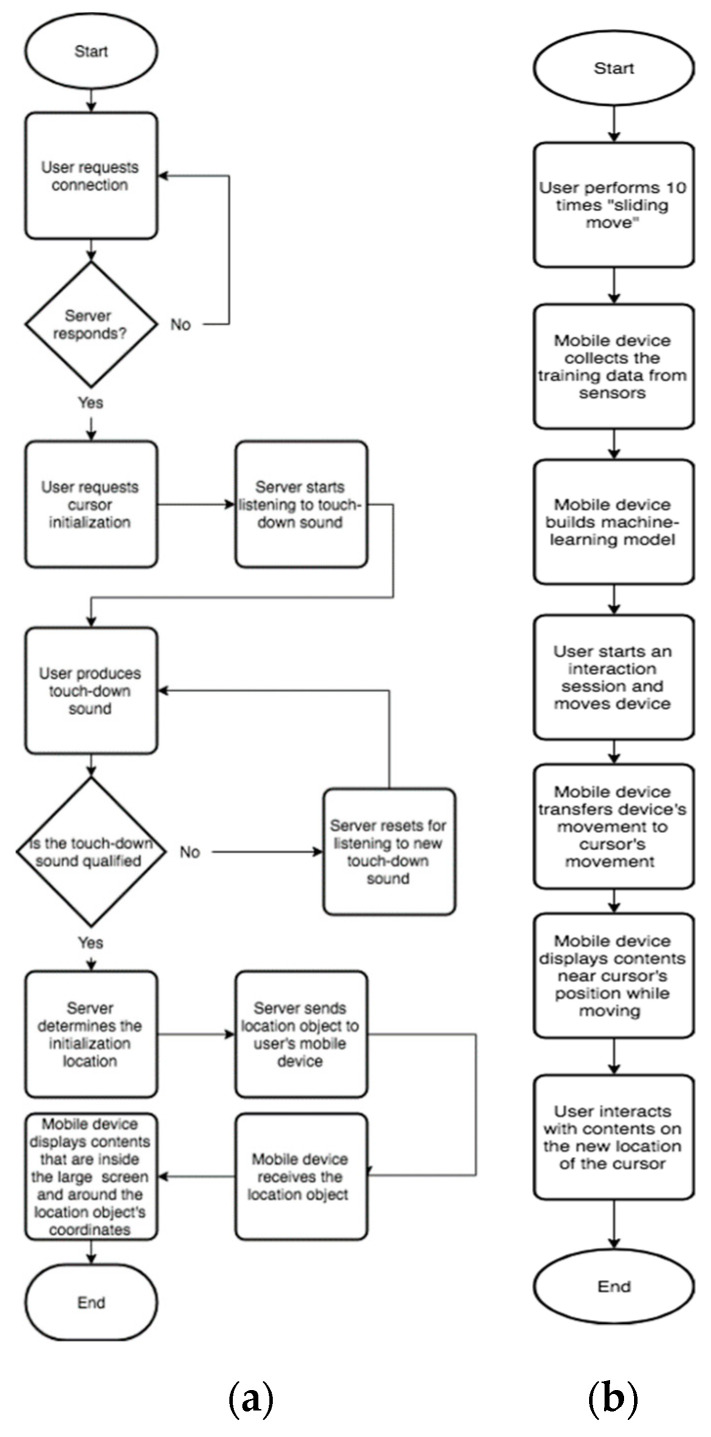
The flowcharts of the cursor initialization and estimation process. (**a**) Cursor initialization; (**b**) cursor motion detection.

The system uses a client-server architecture. The smartphone is the client and the large screen machine is the server. The general working flow is displayed in Figure 2. Figure 2a depicts the cursor initialization process. A user first launches the application from a mobile device and waits for the response from server. The server then will respond. After the mobile device successfully connected to server, the user puts down his/her mobile device on a flat surface and generates a touch-down sound. The touch-down sound is detected by a microphone array that is attached to the large display screen. The server of the large display will check if the touch-down sound is qualified. If it is qualified, it will start the initialization process. If not, the server will wait for a new touch-down sound. Through sound localization, the touch-down location of the mobile device on the flat surface is calculated, which is converted to the initial position of the cursor on a large display. The server sends a location object to the mobile device. Subsequently, the mobile device displays an area of contents on the large screen by using the initial position as the upper left vertex and the physical length and width of the mobile device as the dimension of the content area. Since the large display screen and the mobile device have different screen resolutions, a resolution conversion is applied to ensure that the size of the area cut from the large display is physically equal to the size of the mobile device’s screen.

Figure 2b describes the workflow of cursor estimation. The cursor movement estimation system transfers the mobile device’s 2D movement to the cursor’s 2D movement on the large display. Therefore, we need to detect the motion of the mobile device. There are two critical parameters for motion detection. The first parameter involves the movement status detection, which detects whether the device is moving or not in real time; the second parameter identifies the movement direction. We collect motion data and use machine learning techniques for motion detection. To build a detection model, the user first performs a 14 s training of moving the mobile device, including 2 s of “stand”, 3 s of “move right”, 2 s of “move left”, 2 s of “stand”, 3 s of “move up”, and 2 s of “move down.” We choose these six motions because motion coordinates on a 2D surface can be captured by building a rectangular coordinate system which contains x-axis and y-axis. Thus, any motion on the 2D surface can be represented as movements on x-axis (i.e., “move left” and “move right”) and y-axis (i.e., “move up” and ”move down”).

According to Bohan et al. [25], a typical cursor movement takes 1.002 s to finish and travels 18.75 mm, which is 66 px on the monitor (a 19-inch monitor with a resolution of 1400 × 1050 in their study). One, however, should not simply determine each cursor movement based on the data collected every 1.002 s because the cursor will jump from one location to another rather than moving continuously. Therefore, a typical cursor movement should be broken into pixel-movement detection for classification. We have designed a pixel-movement detection experiments as follows: (1) Collecting the sensors’ raw data during a time interval (0.015 s), which is the time used by the cursor to travel one pixel; (2) Extracting features of the raw data to compose as one sample; (3) six classes are predefined (referred to as “stand_on_x”, “move_right”, “move_left”, “stand_on_y”, “move_up”, and “move_down”) in order to indicate different statuses. (4) The sample is classified into one of the six predefined groups. In this way, both movement status and direction detection can be addressed.

After the movement of mobile device is detected and transferred to a new cursor position, the contents on the mobile device are updated based on the new position as the mobile device moves. The user can directly interact with the large display from the contents on her/his mobile.

## 4. Cursor Initialization

**Figure 3:**
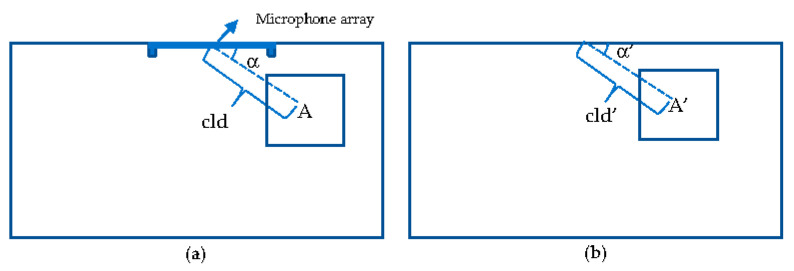
Determine initialization area: (**a**) Large display; (**b**) a working area on a flat surface for the mobile device.

The cursor initialization is achieved by taking advantage of a mobile device, a flat surface (i.e., a table), and a large display equipped with a microphone array. To initialize the cursor, the user first briefly views the content on the large display screen and coarsely selects an area of interest. The user also needs to define a rectangle working area on the flat surface. The working area’s physical dimension is *M* times proportional to the large display screen. The value of *M* is between 0 and 1 since the working area for the mobile device is much smaller and more accessible than the large display screen. After the user decides a desired area *A* on the large display (as in Figure 3a), he or she should estimate the relative position *A’* (in Figure 3b) on the working area based on the center of the microphone array. The physical distance between the center of area *A* and the top center of the large display is cld. The estimated distance cld’on the working area is equal to *M* times cld, and α is equal to α’. 

Then the user produces a touch-down sound by putting the mobile device on the flat hard surface. The sound can be captured by the microphone array. This touch-down sound is then analyzed as the cue for computing the coordinates that represent the location of the sound source. The angle of the sound source aligned to the center of the microphone array can be obtained through the calculation of the time-delay difference between each channel of the microphone array. 

**Figure 4:**
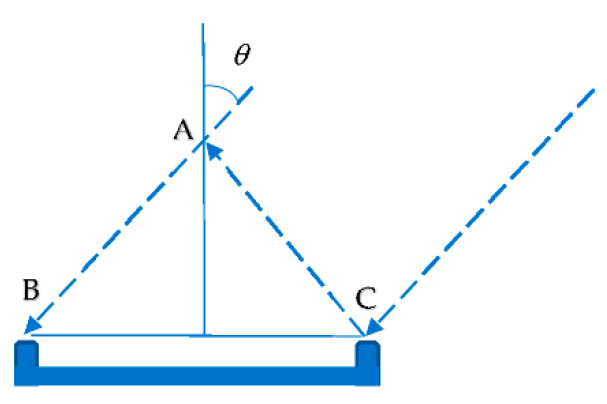
Sound localization schema.

As Figure 4 shows, the distance between A (the sound source) and B (a microphone channel) is evaluated by the production of a time delay difference (TDD) and the speed of the sound. The distance between A and B can be a negative value if the sound source is located on the other side of the microphone array’s central point, and this causes the TDD to be negative. A dual-delay algorithm was implemented with “binaural sound source localization” [26]. The distance between two microphone channels B and C of the microphone array is captured by the measurement of the distance between them. Thus, the angle of the sound source θ can be calculated via Equation (1):

(1) $$\theta =arcsin\frac{AB}{BC}$$

Once the angle has been calculated, the sound intensity can be evaluated through the computation of the decibel level of the sound. Since environmental noise could easily interfere with sound localization, a threshold was proposed to determine whether the sampled sound is a real touch-down sound that is qualified to trigger the initialization. If the intensity of the sampled sound is larger than the threshold, the mobile device is registered as a client of the target large display device. Sound intensity also helps to estimate the relative source sound location (*x, y*), which is implemented in Equation (2):

(2) x=sinθ·dsy=cosθ·ds

In the above formula, ds is an estimated measurement of the distance between the source touch-down sound and the microphone array using sound intensity. The coordinates are used as the relative pixel-based start-point on the target device screen, which takes the center of its top boundary as the original base point. The actual start-point (*X*, *Y*) is estimated based on Equation (3):

(3) $$X=\frac{HR}{2}+x\;Y=0+y$$

where *HR* represents the horizontal resolution of the large display device. However, since θ can vary from −90° to 90° and the value ds is not restricted by the size of the large display screen, it is possible that (*X, Y*) could be out of the range of the large display screen. If either *X* or *Y* is out of the range, then the application should automatically adjust the value to its closest boundary in order to avoid a positioning error. For example, if the large display has a resolution of 1024 px × 768 px and if the source sound coordinates are (500, 866), then the actual location start point is calculated as follows: (024/2 + 500) px, (0 + 866) px. In this case, the system adjusts the value of *Y* from 866 px to 768 px since the vertical positioning is higher than the maximum boundary (866 px > 768 px).

The estimated area is strictly equivalent to the physical size of the mobile device. A key attribute called “dots per inch” (DPI) was used to obtain the relative pixel-based width (*RPW*) and relative pixel-based height (*RPH*) for the mobile device on the large display by means of Equation (4):

(4) $$RPW=\frac{mx}{mdpi}\cdot ldpi\;RPH=\frac{my}{mdpi}\cdot ldpi$$

In the above formula, mx and my are the horizontal and vertical resolutions of the mobile device, and mdpi and ldpi are the DPIs for the mobile device and large display, respectively. Finally, the large display sends a screenshot image and its screen DPI to the mobile device. When the image is received by the mobile device, it only displays the partial area of that screenshot that uses (*X*, *Y*) as the base point and has the width of *RPW* and height of *RPH*. 

## 5. Detecting Cursor Movements

### 5.1. Data Collection

We have predefined six classes (referred to as “stand_on_x”, “move_right”, “move_left”, “stand_on_y”, “move_up”, and “move_down”) to differentiate motion statuses. In order to sample the data for all six predefined class, the pixel-movement classification experiment was designed to have a 7-s data collection session applied on x and y axes respectively, which includes three separate actions: 2 s of “stand”, 3 s of “move_right/up”, and 2 s of “move left/down” on each axis. Table 1 and Table 2 list the detailed actions in the data collection session. The raw data from the accelerometer and gyroscope were sampled at a rate of 590 hertz since, on average, 4131 raw data instances were fetched from the smart phone in 7 s. In addition, the time spent for a 1 px movement was 0.015 s, since in 1.002 s the cursor can travel 66 pixels. 

Moreover, a visual text interface was provided to notify the user of the designated action to perform at a given timestamp. Normally, the visual reaction for a human is 0.25 s [27]. This application notified the user to perform an action 0.25 s before the actual recording time. This adjustment was designed to ensure that the user-recognized timestamp was strictly in accordance with the machine’s recording time.

**Figure 5:**
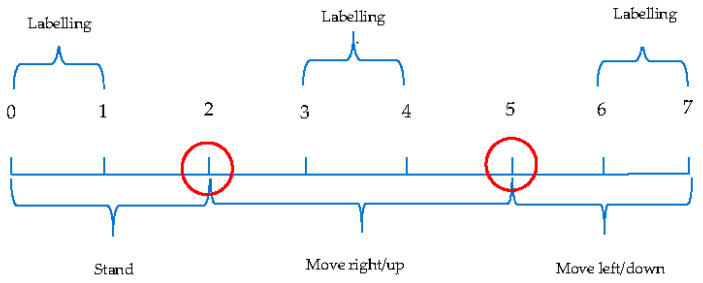
Graph demo of the experiment design.

The time frame as shown in Table 1 and Table 2 was designed due to an important issue encountered during the data collection. This issue raised a question of how to label the benchmark class correctly. Each test may contain thousands of raw instances, and each device has its own mechanical delays because of the variance in the buildup of CPU (central processing unit) speeds. For example, on the threshold of performing actions in Second 2 or Second 5 (indicated by a red circle in Figure 5), the designated class could be mislabeled even when the visual delay time is added prior to the actual action timestamp. For example, presumably, the program labels the raw instances collected after Second 2 as “move right.” However, when the user receives the visual notice and starts to move the device, the timer may have already reached Second 2.2. Thus, the raw instances fetched between Second 2 and Second 2.2 are mislabeled. 

The motivation for the action and labeling design is two-fold. First, sufficient samples for labeling require at least 1 s, which is the typical cursor movement duration (use 1 to replace 1.002 for easy calculation), to be assigned to the labeling session. Additionally, at least 1 s between the labeling session and the action change threshold is also necessary in order to accommodate potential mislabels. Second, it is important to shorten the data collection session as much as possible because this data collection process is required when the user is trying to control the cursor by means of the device’s movements. We aim to maintain a shorter data preprocessing time. If an application has many complex preprocessing steps that consume a substantial amount of time before actual usage, users tend to lose interest in the application. This design allows the experiment simultaneously to maximize the duration of effective sampling data and to minimize the complexity of using the application. Thus, the total time is measured as follows:

Min(tls+tlmp+tlmn+tds+2tdmp+tdmn)

In the above equation, *tls*, *tlmp*, and *tlmn* are the times used for labeling “stand”, “move right/up”, and “move left/down”, while *tds*, *tdmp*, and *tdmn* are required sessions to prevent mislabeling. Since both the labeling session and the mislabel preventing session require 1 s as the minimum time interval, the total required time for data collection is 7 s, based on the above formula. Furthermore, since the action “move right/up” is located in the middle, it required two sessions to prevent mislabeling. The experiment was performed ten times. Five of the tests were left-right-movements, and five of them were up-down movements. 

### 5.2. Data Preprocessing

A preprocessing method was applied to each raw dataset generated from the ten tests. The preprocessing of the raw instances includes two steps. First, only instances with a predefined class label were kept to construct the filtered raw dataset. According to the experiment design, more than half of the raw instances could be potentially labeled incorrectly (1–3 s and 4–6 s in Figure 5); thus, these raw instances were dropped in order to avoid mislabeling. Second, the filtered raw dataset was divided into sub-datasets to facilitate statistical calculations and feature extraction. Nine raw instances were assigned to each sub-dataset sequentially on the time frame so as to test different machine-learning algorithms with the goal of making the application extremely sensitive to the device’s movement detection. Nine raw instances were used as the grouping metrics because a pixel movement typically finished in 0.015 s, and there were, on average, 4131 instances collected in 7 s from each raw dataset. Therefore, to accurately classify each pixel movement, nine raw instances were required. Subsequently, each group of nine raw instances was transformed into a sample that represented a pixel movement, with features calculated by feature-extraction methods. The label values (0, 1, 2, 3, 4, 5) typically represent the device’s statuses (“stand_on_x”, “move_right”, “move_left”, “stand_on_y”, “move_up”, and “move_down”). The label that occurred most frequently in the group were assigned as the label for the corresponding sample. Finally, all the generated samples from a single raw dataset constructed a preprocessed dataset, and ten preprocessed datasets, marked from “data 0” to “data 9”, were produced with, on average, 196 samples in each.

### 5.3. Feature Extraction

A total of 63 features were extracted from the raw data via mathematical or statistical computation. For the raw data, tri-axial accelerometer and gyroscope data, indicated as (ax, ay, az) and (gx, gy, gz), were collected to compute these features. Furthermore, tri-axial speed values (vx, vy, vz) were captured through the use of acceleration and timestamp at each raw instance. Equation (5) depicts the detailed computation method:

(5) $$V_{i}=\{\begin{array}{c} a_{i}\cdot t_{i}\;\;\;\;where\;i=0\\ V_{i-1}+a_{i}\cdot t_{i}\;\;\;\;where\;i>0 \end{array}$$

In general, feature categories can be classified into two domains, represented as the time domain and frequency domain (Table 3). All the 63 extracted features are listed in Table 4.

The frequency-domain features were captured through fast Fourier transform (FFT), which transfers a signal from a time-domain to a frequency-domain [28]. Equation (6) provides:

(6) $$X_{k}=\overset{N\;-\;1}{\underset{n\;=\;0}{\sum }}x_{n}\cdot e^{\;-\;\frac{i2\pi }{N}kn}$$

In this above formula, $x_{n}$ indicates sensors’ readings on time domain, and *N* indicates the length of this signal. The real number part of the computed $X_{k}$ indicates the amplitude spectrum of each frequency domain. The dominant frequency is captured through finding a frequency value that has the maximum amplitude.

The energy of each axis was computed by adding up the square numbers of the raw instances in a signal. The spectral energy was calculated using the same method but with the raw value transformed from a time-domain to a frequency-domain by FFT. Equation (7) (Parseval’s theorem) and (8) [29] describe the detailed calculation:

(7) $$\mathrm{Enery}\left(y\right)=\overset{N}{\underset{n\;=\;1}{\sum }}x_{n}^{2}$$

(8) $$Spectral\left(y\right)=\overset{N}{\underset{n\;=\;1}{\sum }}{\left(FFT\left(x_{n}\right)\right)}^{2}$$

where $x_{n}$ indicates sensors’ readings on time domain, and N indicates the length of this signal.

## 6. Classification and Results

Several studies have been conducted to determine which classification algorithm is the most accurate candidate for extracting patterns from built-in sensors’ data. Algorithms such as the support vector machine (SVM) [30], k-nearest neighbors [31], and naïve Bayes [32] have been used to extract data patterns in order to verify whether daily activities can be detected. In terms of multi-class classification tasks, the study by [33] has suggested that random forest and gradient boosting are the most favored candidates for personal-activity classification. To better fit the data model and classification task in this study, all the classifiers previously mentioned, along with additional candidates, were included in the pool to ascertain whether there were any novel findings.

### 6.1. Basic Cross-Validation

Cross-validation is an evaluation tool that examines whether a model is an effective predictor for data that is completely new and differs from the existing dataset. The simplest way to avoid this “overfit” issue is what is known as a “holdout method.” This method typically splits the dataset into two groups: one group is used for training, and the other group is used for testing. The amount of training and testing is generally assigned at a ratio of 7:3. However, there is an evident weakness that can produce a high variance in the model. The result of each test classification may rely on the endpoint of the training or testing set. Therefore, the strategy of splitting the dataset becomes a critical factor that can affect the evaluation results.

### 6.2. Ten-Folds Cross-Validation

This investigation applied a 10-folds cross-validation to avoid the aforementioned bias. Ten-folds cross-validation is a specific case of a general method known as “K-folds cross-validation.” Using this K-folds cross-validation, the dataset could be split into K subsets, and on each subset the holdout method was performed once. Each iteration only used one of the K subsets as the test group and the other K-1 subsets as the training group. This method notably improved the holdout since it mitigated the impact of the data-division strategy. As the K value increased, the variance in the evaluation results declined. Moreover, K = 10 was used because the number of test times in this study equals 10. Thus, to keep the consistency, the authors chose 10 as the value for parameter K.

**Figure 6:**
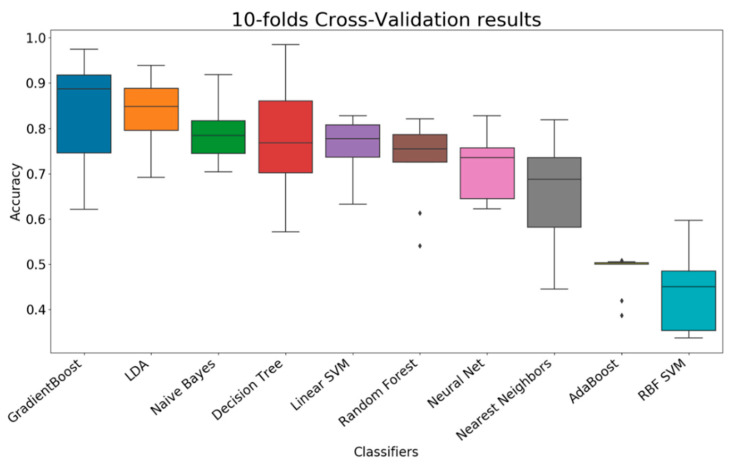
Cross-validation results for all features (box plot).

A 10-folds cross-validation was applied on a dataset that contained 1969 samples. This dataset was combined by appending ten preprocessed datasets together. Ten classifiers were evaluated by the accuracy performance (Figure 6 and Table 5); these classifiers included AdaBoost (adaptive boosting), decision tree, gradient boosting, LDA (linear discriminant analysis), linear SVM (support vector machine), naïve Bayes, nearest neighbors, neural network, random forest, and RBF (radial basis function) SVM. 

In Figure 6, from left to right, the performance of all classifiers is sorted by descending order of mean and by ascending order of standard deviation. From the observations, gradient boosting, LDA, and naïve Bayes are the top three classifiers, which have reached the average accuracy of 83.65%, 83.43%, and 79.42%; their standard deviation is measured at 11.18%, 7.43%, and 6.68%, respectively. Based on the average accuracy, gradient boosting should be selected as the agent for this classification problem; however, gradient boosting has a higher standard deviation (11.18% versus 7.43%) but a similar average accuracy (83.65% versus 83.43%) compared with LDA. This comparison suggests that the performance of gradient boosting may significantly vary when different training and testing data are used for classification problems. Thus, LDA is recommended when all features are included.

In machine learning, besides the accuracy rate, the manner in which the results of the error predictions are distributed is also critical for downstream analysis. The distribution of errors can be determined through an examination of the confusion matrix of the classification results proposed by each classifier. In this work, the confusion matrices of the top three classifiers (gradient boosting, LDA, naïve Bayes) were demonstrated (Table 6).

The confusion matrix generally shows the distribution of correct and error predictions. Each row label indicates the predicted class, and the column label indicates the actual class. For example, based on the confusion matrix of naïve Bayes, the first observation of 206 falls under the column of “stand_on_x” and the row of “stand_on_x” as well. This result means that 206 samples are predicted as “stand_on_x” and also belong to the “stand_on_x” class, and this means, ultimately, that these 206 samples have been predicted correctly. However, the second horizontal observation 2 indicates that two samples are predicted as “stand_on_x” but actually belong to the “move_right” class; this means that these samples have been predicted incorrectly.

Some notable findings can be ascertained from the confusion matrix. First, it seems that all these three classifiers have managed an exemplary performance in distinguishing “move” and “stand” regardless of direction since there are few observations in the cells of “stand_on_x/y” that are classified as “move_right/left/up/down.” Second, commonly, samples are classified into incorrect labels where the difference only lies in an axis (x or y) compared with the original label. For example, there are many error predictions that fail to distinguish whether the sample is in the “stand_on_x” group or the “stand_on_y” group.

## 7. Feature Selection

In machine learning, feature selection plays an important role that can substantially impact not only the learning accuracy of the prediction model but also the efficiency and user experience of the application. Feature selection represents the process of fetching a subset that contains the most relevant features from an original feature set based on statistical algorithms and has been proven to be accurate through both theoretical and practical success in multiple application scenarios [34,35]. To determine what features should be selected, multiple methods were applied in our work, and these methods can be grouped into two categories: algorithm-based methods and manual feature selection methods.

### 7.1. Algorithm-Based Methods

Three algorithm-based methods were tested in this paper: linear correlation analysis, select k best, and recursive feature elimination (RFE). All were evaluated by applying 10-folds cross-validation on the feature sets proposed.

#### 7.1.1. Linear Correlation Analysis

Linear correlation is a statistical method to investigate the strength of association between two features in order to obtain the most relevant features and to remove irrelevant features through an examination of the strength between each feature and the labeled class. Moreover, a Pearson correlation coefficient was computed for each pair of features by means of Equation (9):

(9) $$r=\frac{\sum_{i}(x_{i}-\bar{x})\left((y_{i}-\bar{y}\right)}{\sqrt{\sum_{i}{\left(x_{i}-\bar{x}\right)}^{2}}\sqrt{\sum_{i}{\left(y_{i}-\bar{y}\right)}^{2}}}$$

In this formula, $x_{i}$ and $y_{i}$ represent the values of two features, and $\bar{x}$ and $\bar{y}$ are the mean values of each feature. The result is always a decimal number between –1 and 1. If this number is close to 1, then the two variables X and Y reveal a high positive correlation. If this number is close to –1, then the two variables reveal a high negative correlation. A threshold of 0.5 was proposed [36] so as to keep features that have absolute values of their correlations with a label class larger or equal to 0.5. A feature set containing four features was generated: “mean*vz”, “median*vz”, “energy*vz”, and “spectral_energy*vz”. 

#### 7.1.2. Select K Best

A select K best method uses a specific function to score each feature and to select the highest K scoring features. We computed an analysis of variance (ANOVA) F-value between the label and each feature, and it used K = 10 to perform this task because the author aimed to investigate the performance of each classifier when the number of features increased compared with a linear correlation analysis. Selected features were as follows: “median*vz”, “mean*vz”, “spectral_energy*vz”, “energy*vz”, “spectral_energy*vy”, “energy*vy”, “spectral_energy*vx”, “energy*vx”, “median*vy”, and “mean*vy”.

#### 7.1.3. Recursive Feature Elimination

Recursive feature elimination (RFE) is a method that proposes certain candidate features by gradually focusing on a smaller set of features. Usually, it starts with a trained estimator to assign an importance value to each feature, and then the feature with the lowest importance value is eliminated from the candidate pool. This process is continued recursively until the desired number of features has been satisfied.

This study employed a linear SVM as a trained estimator since it has a high accuracy and an efficient generalization ability for removing features recursively [37]. With this algorithm fitted into the dataset, the RFE model proposed an optimized number of 31 features.

#### 7.1.4. Algorithm-Based Results

Cross-validation results for the top three classifiers in each method have been demonstrated in Table 7. From the observation of proposed features, there are 33 unique features selected by all three methods, and 84.85% (28 out of 33) are time-domain-related; this potentially suggests that frequency-domain features are not as important as time-domain features for this classification task. In addition, LDA, naïve Bayes, and gradient boosting are potential candidates to be recognized as the best classifier for detecting device movement since they are ranked as the top three classifiers in at least two feature-selection methods. Furthermore, 39.4% (13 out of 33) of the proposed features are speed-related, and both the select K best and the linear correlation selected features that are all speed-related; this may indicate that speed is a critical vector in terms of determining device movement.

### 7.2. Manual Feature Selection

Manual feature selection is another method for pursuing a small set of features by removing irrelevant features. However, instead of using algorithms to determine feature importance, Manual feature selection proposes feature sets based on assumptions that may explain what features are related to the success of movement detection. For example, in physics, speed typically describes where and how fast the object is moving. Angular velocity detects whether the object has any rotation event. Thus, an assumption can be made as when the machine-learning model tries to detect the four-direction movement of a mobile device, it is better using speed-related features rather than angular velocity-related features to build the model. Since the key of determining four-direction movement is to detect where and how fast the object is moving. Movements on a flat surface barely produce rotation event. 

Another hypothesis is that speed-related features should exclude standard deviation and minimum-maximum difference in order to have a better performance. Since the standard deviation and minimum-maximum difference can only measure the extent of speed alteration rather than indicate where and how fast the object is moving. The ideal feature category should be mean or median because they reflect the raw values of speed in a pixel-movement time interval. 

The core process of manual feature selection is composed of five steps: (1) Evaluating the classification performance of feature sets that each of them relates to either one vector (acceleration, angular velocity, and speed) or one feature category (mean, std, min_max_gap, median, energy, dominant frequency, and spectral energy). Since each vector or feature category has its unique physical or statistical meanings, it is better to understand how they uniquely impact the success of the classification. (2) Finding out the vector VH that its related features can produce a higher recognition rate than other vectors. (3) Finding out the feature category FCH that its related features can produce a higher recognition rate than other feature categories. (4) Selecting a combined feature set that contains features relates to VH and FCH simultaneously. (5) Evaluating the performance of this combined feature set to verify if the recognition rate can be higher than former feature sets. The objective of this section is to examine whether using features that relate simultaneously to the most relevant vector and most relevant feature category can reach a higher performance

#### 7.2.1. Vector-Based Feature Selection

The difference between vectors acts as an imperative factor for the success of the classification task because different vectors represent their own unique physical significance. Therefore, it is critical to evaluate features related to a single vector or a single feature category separately. An analysis for selecting features based on different vectors is demonstrated in Table 8.

From the results of vector-based feature selection, it is obvious that as long as speed-related features are included, then the average accuracy of classification is always significantly higher (80.47% > 58.16%) than the feature set without speed-related features. At the same time, acceleration- or angular velocity-related features can be considered as artifacts for the classification task: first, because features based on these two vectors are performing at an extremely low accuracy rate (on average, 58.16%); second, because there is no significant accuracy improvement when either acceleration- or angular velocity-related features are mixed with speed-related features (for example, LDA sits at 80.03% with speed-only features, but at 81.71% and 82.31% when mixing speed with acceleration- and angular velocity features, respectively). 

#### 7.2.2. Feature-Category-Based Feature Selection

The feature categories presented in the feature extraction section were inspired by previous studies [38]. However, some of the feature categories are either mathematical- or statistical-confounding factors for the classification problem. Thus, it is wise to analyze them separately in order to observe what feature categories contribute positively to the machine-learning model.

This study applied cross-validation on seven feature categories independently, and the results, including the top three classifiers that have the highest accuracy rates, are shown in Table 9. From the observations, it seems that through the application of median-related features, the accuracy rate can boom to 85.36% when naïve Bayes is used as the classifier. However, there is another observation that also uses naïve Bayes as the classifier but applies mean-related features; this reaches a similar recognition rate of 85.25%. These findings suggest that either median- or mean-related features contribute to the learning process. In addition, the accuracy rate of main-frequency-related (“main_freq”) features are significantly lower than the accuracy of other feature categories; this could indicate that major frequency-related features are irrelevant to the success of the classification task.

#### 7.2.3. Combined Analysis

From the brief analysis of the previous two feature-selection methods, another hypothesis can be posited that if the feature set was downsized into a state where only speed and mean- or median-related features were included, then the accuracy of the specific classifier should be higher or remain at the same level. To verify this assumption, this study has created two feature sets: one containing only the speed and mean-related feature, and the other only the speed and median-related feature. Cross-validation was then applied on these two feature sets. After generating the results, the author selected “gradient boosting”, “LDA”, and “naïve Bayes” as the benchmark classifiers for comparing since these are the most recurrent classifiers considered to be high performing, based on the previous results. 

The compared results (Table 10) provide strong evidence for declining the aforementioned hypothesis. With a focus on features that are only related to speed and mean, the performance of the classification actually decreased for all three benchmark classifiers, compared to the performance of the feature set with all vectors (speed, acceleration, and angular velocity). The same trend occurred on another feature set (speed and median related feature) since the recognition rates of benchmark classifiers boomed when features related to all vectors and the median were involved in the learning model. 

Moreover, these results also show that acceleration- and angular velocity-related features cannot contribute to the performance of the classification task because it is evident that the addition of more vector-based features can lead to a boom in performance (gradient boosting: from 79.10 to 84.00%; LDA: from 72.34 to 77.98%; naïve Bayes: from 74.38 to 85.25%).

Furthermore, this performance boom was not driven from the addition of the number of features: Table 10 shows that even with 21 features or all 63 features selected, gradient boosting and naïve Bayes performed worse than previous feature sets (all vectors and the mean, all vectors and the median) that only have nine features.

## 8. Conclusions and Future Work

In this paper, we have proposed a low-cost, intuitive, and physically unconstrained cursor position system. The user can conveniently browse and interact with a large display device at a distance from her/his mobile device. We use a sound localization technique to initiate the cursor’s starting position on a large display device, which enables the initial connection between a mobile device and a large display screen. A novel data collection framework has been implemented that helps the supervised model to avoid mislabeling for training sets when working with very sensitive sensors, in which a data-analysis pipeline is built for characterizing data into statistical features (mean, standard deviation, min-max difference, and power energy) and spectral features (dominant frequency and spectral energy). A comprehensive study of different machine-learning algorithms and feature selection sets is conducted in the experiment. In total, 63 features and 10 classifiers have been employed to construct the machine-learning models. Multiple feature-selection methods have been applied to find an optimized machine-learning model. The study shows that naïve Bayes, gradient boosting, and LDA as the reliable classifiers to build machine-learning models for detecting motion of mobile devices. 

In future, we plan to extend our work to other mobile devices, such as tablets and smartwatches. Current generations of mobile devices already have built-in accelerometers and our approach does not need any additional hardware. In the experiments, we use a smartphone with Android OS. It is feasible to apply our system to other devices using the same operating system. Our future goal is to create a cross-platform application that can run the application on iOS devices as well. Although we have tested the system and achieved a seamless smooth interaction with the large display screen as the mobile device moves, we plan to conduct a user study to evaluate the performance of our system and gather more feedback to improve the current design.

## Figures and Tables

**Table 1:** Action design.

Actions	Timestamp
Stand	0–2 s
Move right	2–5 s
Move left	5–7 s
Stand	0–2 s
Move up	2–5 s
Move down	5–7 s

**Table 2:** Labeling design.

Labels	Timestamp
0(stand_on_x)	0–1 s
1(move_right)	3–4 s
2(move_left)	6–7 s
3(stand_on_y)	0–1 s
4(move_up)	3–4 s
5(move_down)	6–7 s

**Table 3:** Extracted features on domains.

Domains	Feature Categories
Time domain	Mean
	Standard deviation
	Minimum-maximum difference
	Median
	Energy
Frequency domain	Dominant frequency
	Spectral energy

**Table 4:** All 63 features.

Vectors	Axes	Mean	Standard Deviation	Minimum-Maximum Difference	Median	Energy	Dominant Frequency	Spectral Energy
**Acceleration**	X	mean*ax	std*ax	min_max_gap*ax	median*ax	energy*ax	main_freq*ax	spectral_energy*ax
	Y	mean*ay	std*ay	min_max_gap*ay	median*ay	energy*ay	main_freq*ay	spectral_energy*ay
	Z	mean*az	std*az	min_max_gap*az	median*az	energy*az	main_freq*az	spectral_energy*az
**Rotation**	X	mean*gx	std*gx	min_max_gap*gx	median*gx	energy*gx	main_freq*gx	spectral_energy*gx
	Y	mean*gy	std*gy	min_max_gap*gy	median*gy	energy*gy	main_freq*gy	spectral_energy*gy
	Z	mean*gz	std*gz	min_max_gap*gz	median*gz	energy*gz	main_freq*gz	spectral_energy*gz
**Speed**	X	mean*vx	std*vx	min_max_gap*vx	median*vx	energy*vx	main_freq*vx	spectral_energy*vx
	Y	mean*vy	std*vy	min_max_gap*vy	median*vy	energy*vy	main_freq*vy	spectral_energy*vy
	Z	mean*vz	std*vz	min_max_gap*vz	median*vz	energy*vz	main_freq*vz	spectral_energy*vz

**Table 5:** Cross-validation results for all features.

Classifiers	Accuracy Mean	Accuracy Std
Gradient boosting	83.65%	11.18%
LDA	83.43%	7.43%
naïve Bayes	79.42%	6.68%
Decision tree	76.91%	11.66%
Linear SVM	76.24%	5.60%
Random forest	72.70%	6.22%
Neural net	72.47%	7.06%
Nearest neighbors	66.51%	11.05%
AdaBoost	48.24%	4.03%
RBF SVM	44.27%	8.66%

**Table 6:** Confusion matrices for top three classifiers.

**Gradient Boosting**						
stand_on_x	move_right	move_left	stand_on_y	move_up	move_down	**Classified as**
208	1	0	116	0	0	**stand_on_x**
0	277	14	0	38	0	**move_right**
0	1	311	0	5	19	**move_left**
70	1	0	261	0	0	**stand_on_y**
0	26	3	0	289	0	**move_up**
0	0	22	0	0	307	**move_down**
**LDA**						
stand_on_x	move_right	move_left	stand_on_y	move_up	move_down	**Classified as**
218	0	0	107	0	0	**stand_on_x**
0	307	0	0	22	0	**move_right**
0	6	307	0	18	5	**move_left**
127	1	0	204	0	0	**stand_on_y**
0	29	9	1	279	0	**move_up**
0	0	0	0	1	328	**move_down**
**naïve Bayes**						
stand_on_x	move_right	move_left	stand_on_y	move_up	move_down	**Classified as**
206	2	2	115	0	0	**stand_on_x**
0	242	23	0	64	0	**move_right**
0	16	252	0	52	16	**move_left**
73	3	0	255	1	0	**stand_on_y**
0	3	19	0	296	0	**move_up**
0	2	14	0	0	313	**move_down**

**Table 7:** Cross-validation for algorithm-based feature selection.

Feature Selection Method	Selected Features	Classifiers	Accuracy_Mean	Accuracy_Std
Linear correlation	“mean*vz”, “median*vz”, “energy*vz”, “spectral_energy*vz”	naïve Bayes	73.20%	12.07%
		RBF SVM	72.57%	11.50%
		Neural Nnet	72.13%	13.20%
Select k best (Anova)	“median*vz”, “mean*vz”, “spectral_energy*vz”, “energy*vz”, “spectral_energy*vy”, “energy*vy”, “spectral_energy*vx”, “energy*vx”, “median*vy”, “mean*vy”	LDA	78.75%	8.13%
		Random forest	76.26%	9.59%
		Gradient boosting	76.12%	10.81%
Recursive feature elimination	“mean*ax”, “mean*ay”, “mean*az”, “mean*vx”, “mean*vy”, “mean*vz”, “mean*gx”, “mean*gz”, “min_max_gap*ax”, “min_max_gap*ay”, “min_max_gap*az”, “min_max_gap*vy”, “min_max_gap*gy”, “min_max_gap*gz”, “median*ax”, “median*ay”, “median*az”, “median*vx”, “median*vy”, “median*vz”, “median*gz”, “main_freq*gy”, “main_freq*gz”, “energy*ax”, “energy*az”, “energy*vx”, “energy*vz”, “energy*gy”, “energy*gz”, “spectral_energy*vy”, “spectral_energy*vz”	Gradient boosting	84.07%	10.74%
		LDA	82.82%	8.14%
		naïve Bayes	80.40%	4.26%

**Table 8:** Performance of vector-based feature selection.

Vector Selection	Selected Features	Number of Features	Classifiers	Accuracy_Mean	Accuracy_Std
Acceleration	“mean*ax”, “mean*ay”,”mean*az”,”std*ax”, “std*ay”,”std*az”, “std*az”,”min_max_gap*ax”, “min_max_gap*ay”,”min_max_gap*az”,”median*ax”, “median*ay”,”median*az”,”energy*ax”, “energy*ay”,”energy*az”, “main_freq*ax”, “main_freq*ay”,”main_freq*az”,”spectral_energy*ax”, “spectral_energy*ay”,”spectral_energy*az”	21	Gradient boosting	63.87%	5.27%
			Random forest	61.07%	5.26%
			Decision tree	57.12%	4.28%
Angular velocity	“mean*gx”, “mean*gy”,”mean*gz”,”std*gx”, “std*gy”,”std*gz”, “std*gz”,”min_max_gap*gx”, “min_max_gap*gy”,”min_max_gap*gz”,”median*gx”, “median*gy”,”median*gz”,”energy*gx”, “energy*gy”,”energy*gz”, “main_freq*gx”, “main_freq*gy”,”main_freq*gz”,”spectral_energy*gx”, “spectral_energy*gy”,”spectral_energy*gz”	21	Decision tree	51.96%	5.31%
			Gradient boosting	51.96%	3.97%
			Random forest	50.67%	7.45%
Speed	“mean*vx”, “mean*vy”,”mean*vz”,”std*vx”, “std*vy”,”std*vz”, “std*vz”,”min_max_gap*vx”, “min_max_gap*vy”,”min_max_gap*vz”,”median*vx”, “median*vy”,”median*vz”,”energy*vx”, “energy*vy”,”energy*vz”, “main_freq*vx”, “main_freq*vy”,”main_freq*vz”,”spectral_energy*vx”, “spectral_energy*vy”,”spectral_energy*vz”	21	LDA	80.03%	9.84%
			Random forest	79.98%	8.92%
			Gradient boosting	79.06%	6.92%
Acceleration and angular velocity	“mean*gx”, “mean*gy”,”mean*gz”,”std*gx”, “std*gy”,”std*gz”, “std*gz”,”min_max_gap*gx”, “min_max_gap*gy”,”min_max_gap*gz”,”median*gx”, “median*gy”,”median*gz”,”energy*gx”, “energy*gy”,”energy*gz”, “main_freq*gx”, “main_freq*gy”,”main_freq*gz”,”spectral_energy*gx”, “spectral_energy*gy”,”spectral_energy*gz”,”mean*gx”, “mean*gy”,”mean*gz”,”std*gx”, “std*gy”,”std*gz”, “std*gz”,”min_max_gap*gx”, “min_max_gap*gy”,”min_max_gap*gz”,”median*gx”, “median*gy”,”median*gz”,”energy*gx”, “energy*gy”,”energy*gz”, “main_freq*gx”, “main_freq*gy”,”main_freq*gz”,”spectral_energy*gx”, “spectral_energy*gy”,”spectral_energy*gz”	42	Gradient boosting	65.08%	5.33%
			Random forest	62.20%	3.51%
			Decision Tree	59.59%	5.95%
Speed and acceleration	“mean*ax”, “mean*ay”,”mean*az”,”std*ax”, “std*ay”,”std*az”, “std*az”,”min_max_gap*ax”, “min_max_gap*ay”,”min_max_gap*az”,”median*ax”, “median*ay”,”median*az”,”energy*ax”, “energy*ay”,”energy*az”, “main_freq*ax”, “main_freq*ay”,”main_freq*az”,”spectral_energy*ax”, “spectral_energy*ay”,”spectral_energy*az”,”mean*vx”, “mean*vy”,”mean*vz”,”std*vx”, “std*vy”,”std*vz”, “std*vz”,”min_max_gap*vx”, “min_max_gap*vy”,”min_max_gap*vz”,”median*vx”, “median*vy”,”median*vz”,”energy*vx”, “energy*vy”,”energy*vz”, “main_freq*vx”, “main_freq*vy”,”main_freq*vz”,”spectral_energy*vx”, “spectral_energy*vy”,”spectral_energy*vz”	42	Gradient boosting	83.25%	10.90%
			naïve Bayes	83.01%	7.36%
			LDA	81.71%	7.05%
Speed and angular velocity	“mean*vx”, “mean*vy”,”mean*vz”,”std*vx”, “std*vy”,”std*vz”, “std*vz”,”min_max_gap*vx”, “min_max_gap*vy”,”min_max_gap*vz”,”median*vx”, “median*vy”,”median*vz”,”energy*vx”, “energy*vy”,”energy*vz”, “main_freq*vx”, “main_freq*vy”,”main_freq*vz”,”spectral_energy*vx”, “spectral_energy*vy”,”spectral_energy*vz”,”mean*gx”, “mean*gy”,”mean*gz”,”std*gx”, “std*gy”,”std*gz”, “std*gz”,”min_max_gap*gx”, “min_max_gap*gy”,”min_max_gap*gz”,”median*gx”, “median*gy”,”median*gz”,”energy*gx”, “energy*gy”,”energy*gz”, “main_freq*gx”, “main_freq*gy”,”main_freq*gz”,”spectral_energy*gx”, “spectral_energy*gy”,”spectral_energy*gz”	42	LDA	82.31%	8.94%
			Gradient boosting	81.75%	7.76%
			Linear SVM	75.71%	6.55%

**Table 9:** Performance of feature-category based feature selection.

Feature-Category	Selected Features	Number of Features	Classifiers	Accuracy_Mean	Accuracy_Std
Mean	“mean*ax”,”mean*ay”,”mean*az”,”mean*gx”,”mean*gy”,”mean*gz”,”mean*vx”,”mean*vy”,”mean*vz”	9	naïve Bayes	85.25%	6.53%
			Gradient boosting	84.00%	12.42%
			LDA	77.98%	11.57%
Std	“std*ax”,”std*ay”,”std*az”,”std*gx”,”std*gy”,”std*gz”,”std*vx”,”std*vy”,”std*vz”	9	Gradient boosting	56.51%	4.01%
			Decision tree	48.54%	6.84%
			Random forest	46.51%	6.40%
Min_max_gap	“min_max_gap*ax”,”min_max_gap*ay”,”min_max_gap*az”,”min_max_gap*gx”,”min_max_gap*gy”,”min_max_gap*gz”,”min_max_gap*vx”,”min_max_gap*vy”,”min_max_gap*vz”	9	Gradient boosting	58.05%	4.26%
			Decision tree	50.29%	5.56%
			Random forest	50.27%	4.88%
Median	“median*ax”,”median*ay”,”median*az”,”median*gx”,”median*gy”,”median*gz”,”median*vx”,”median*vy”,”median*vz”	9	naïve Bayes	85.36%	7.27%
			Gradient boosting	82.54%	10.69%
			Random forest	78.55%	7.82%
Energy	“energy*ax”,”energy*ay”,”energy*az”,”energy*gx”,”energy*gy”,”energy*gz”,”energy*vx”,”energy*vy”,”energy*vz”	9	Gradient boosting	83.00%	11.06%
			naïve Bayes	78.50%	6.23%
			Decision tree	77.93%	11.29%
Main_freq	“main_freq*ax”,”main_freq*ay”,”main_freq*az”,”main_freq*gx”,”main_freq*gy”,”main_freq*gz”,”main_freq*vx”,”main_freq*vy”,”main_freq*vz”	9	LDA	25.95%	1.60%
			Linear SVM	25.85%	0.72%
			Decision tree	25.49%	1.06%
Spectral_energy	“spectral_energy*ax”,”spectral_energy*ay”,”spectral_energy*az”,”spectral_energy*gx”,”spectral_energy*gy”,”spectral_energy*gz”,”spectral_energy*vx”,”spectral_energy*vy”,”spectral_energy*vz”	9	Gradient boosting	83.10%	11.42%
			Decision tree	78.42%	11.84%
			naïve Bayes	78.29%	7.19%

**Table 10:** Feature selection performance with benchmark classifiers.

Description	Feature Set	Number of Features	Gradient Boosting	LDA	Naïve Bayes
Speed and mean	“mean*vx”,”mean*vy”,”mean*vz”	3	79.10%	72.34%	74.38%
All vectors and mean	mean*vx,”mean*vy”,”mean*vz”,”mean*ax”,”mean*ay”,”mean*az”,”mean*gx”,”mean*gy”,”mean*gz”	9	84.00%	77.98%	85.25%
Speed and median	“median*vx”,”median*vy”,”median*vz”	3	78.32%	72.34%	74.38%
All vectors and median	“median*vx”,”median*vy”,”median*vz”,”median*ax”,”median*ay”,”median*az”,”median*gx”,”median*gy”,”median*gz”	9	82.54%	77.88%	85.36%
Speed and all feature categories	mean*vx, mean*vy”,”mean*vz”,”std*vx”, “std*vy”,”std*vz”,”min_max_gap*vx”, “min_max_gap*vy”,”min_max_gap*vz”,”median*vx”, “median*vy”,”median*vz”,”energy*vx”, “energy*vy”,”energy*vz”, “main_freq*vx”, “main_freq*vy”,”main_freq*vz”,”spectral_energy*vx”, “spectral_energy*vy”,”spectral_energy*vz”	21	79.06%	80.03%	71.43%
All 63 features	All 63 features	63	83.65%	83.43%	79.42%

## Data Availability

Data are available upon request from the authors.
